# The Mental Health of Elite-Level Coaches: A Systematic Scoping Review

**DOI:** 10.1186/s40798-023-00655-8

**Published:** 2024-02-11

**Authors:** Joshua Frost, Courtney C. Walton, Rosemary Purcell, Krista Fisher, Kate Gwyther, Michael Kocherginsky, Simon M. Rice

**Affiliations:** 1 Centre for Youth Mental Health, The University of Melbourne, Melbourne, Australia; 2 Elite Sports and Mental Health, Orygen, Melbourne, Australia; 3 Melbourne School of Psychological Sciences, The University of Melbourne, Melbourne, Australia

**Keywords:** Coaches, High-performance, Sports psychology, Wellbeing, Coaching effectiveness

## Abstract

**Background:**

Elite-level coaches are exposed to multiple performance, organisational and personal stressors which may contribute to reduced mental health and wellbeing. This systematic scoping review examined the current body of evidence to explore what is known about the mental health of elite-level coaches (i.e. wellbeing and mental ill-health), the risk and protective factors that influence coach mental health, and the relationship between mental health and coaching effectiveness.

**Methods:**

The review adhered to the Preferred Reporting Items for Systematic reviews and Meta-Analyses extension for Scoping Reviews (PRISMA-ScR) guidelines. A systematic search was undertaken and updated in September 2022 using six electronic databases.

**Results:**

12,376 studies were identified and screened, with 42 studies satisfying the inclusion criteria. Despite the paucity of high-quality research, findings indicated that 40% of the included studies examined themes connected to wellbeing, with 76% assessing the nature or prevalence of mental ill-health in elite-level coaches. Among studies exploring mental ill-health, coach burnout was the primary focus, while scant research examined symptoms associated with clinical disorders (e.g. anxiety and depression). Overall, psychological outcomes for elite-level coaches were shaped by risk and protective factors operating at the individual, interpersonal, organisational and societal level. Preliminary evidence was also found to suggest that poor mental health may contribute to reduced coaching effectiveness. It is proposed that coaching effectiveness could therefore be employed as a ‘hook’ to engage elite-level coaches in greater consideration of their mental health needs.

**Conclusion:**

Alongside the development of methodologically robust research, there is a need to examine dynamic individual (e.g. psychological skills), interpersonal (e.g. strong social supports) and organisational (e.g. workload) factors that aim to preserve the mental health and optimise the efficacy of elite-level coaches.

**Supplementary Information:**

The online version contains supplementary material available at 10.1186/s40798-023-00655-8.

## Key Points

- At present, there is lack of high-quality research investigating the mental health of elite-level coaches. Limited research has also focused on understanding the nature and prevalence of mental disorders in elite-level coaches, with greater attention directed towards themes associated with wellbeing and burnout.
- Current evidence indicates that a variety of risk and protective factors operating at the individual, interpersonal, organisational and societal level may influence the mental health of elite-level coaches. Further research is required to determine which factors contribute most significantly towards mental health outcomes.
- Despite the paucity of research, preliminary evidence indicates that mental health may impact coaching effectiveness, including both a coach’s own and team/athlete’s functioning. Robust research is needed to examine this relationship in greater depth, to assess whether coaching effectiveness could be employed as a ‘hook’ to engage elite-level coaches in greater consideration of their mental health needs.

**Supplementary Information:**

The online version contains supplementary material available at 10.1186/s40798-023-00655-8.

## Background

Coaches who operate in elite and professional sports encounter a range of stressors that have the potential to influence or compromise mental health. These demands emanate from the diverse set of roles and responsibilities elite-level coaches are required to perform and fulfil. In addition to their technical, tactical, and strategic expertise, coaches also serve as educators, motivators, counsellors and even friends [1–4]. Despite the perception that coaches primarily shape the performing outcomes of an athlete or sports team, researchers have proposed that elite-level coaches should also be considered as performers in their own right (e.g. competition preparation and psychological state) [5, 6]. On top of performance pressures, elite-level coaches also encounter multiple organisational challenges, including long working hours, job insecurity, media scrutiny and pressures to satisfy board/management expectations [1, 6–9]. Similarly, coaches are also confronted with various personal stressors, including social isolation and relationship issues [4, 6, 10–12]. Much like the elite athletes they coach, this combination of performance, organisational, and personal-related stressors may contribute to a coach’s experience of mental health. With exposure to these multiple stressors, it is critical to ensure that coaches possess appropriate coping or stress management strategies, in conjunction with robust social networks and organisational supports that look to protect and preserve the mental health of elite-level coaches [13, 14].

Over the past decade, research into the mental health of elite sportspeople has gathered considerable momentum. To date, research has primarily examined the mental health outcomes of elite athletes [15–18], with comparatively less research focusing on understanding these psychological experiences among coaches [19]. This is significant as coaches operate in the same elite-level environments as athletes, but arguably possess a greater set of performance and organisational responsibilities, given they are often expected to manage and oversee the performances of multiple athletes [7], whilst simultaneously acting as the public face and cultural identity of a sporting organisation [1]. Although coaches who operate within community and recreational settings encounter a variety of stressors that may threaten an individual’s mental health (e.g. parental pressures, lack of participation) [20], elite-level coaches generally operate within high-pressure environments, where the margin between success and failure may be scrutinised by a range of individuals who operate within the public domain (e.g. fans, the media, former elite sportspeople) [21, 22], and can negatively impact one’s employment status if a coach is perceived to have regularly underperformed [23, 24]. The mental health experiences and literacy of elite-level coaches should therefore not be overlooked or underestimated. Moreover, given these individuals hold prominent leadership roles, coaches play a role in cultivating an organisational or team environment that facilitates optimal wellbeing [25]. This may include a decisive role in shaping the cultural and institutional attitudes towards mental health in elite sports organisations (e.g. help-seeking) [26, 27].

The evolving fields of sports psychology and psychiatry have operationalised mental health in myriad ways. Keyes' [28, 29] dual-continua model of mental health has emerged as a prominent theoretical framework throughout elite sport [30, 31]. This framework employs a bivariate conceptualisation to assert that mental health should be perceived as a complete state comprising of two distinct but related phenomena: mental wellbeing and mental ill-health. For instance, the model’s first dimension is characterised by an individual’s level of mental wellbeing. This dimension broadly reflects the experience of positive feelings and happiness (i.e. hedonic wellbeing), as well as the satisfaction and perception of purpose within one’s life (i.e. eudaimonic wellbeing). This dimension can be generally determined by a combination of emotional (e.g. affective states), psychological (e.g. individual functioning) and social (e.g. societal functioning) wellbeing [32]. Keyes’ model postulates that these three factors determine whether an individual is experiencing high (flourishing), moderate or low (languishing) mental wellbeing. In contrast, the model’s second dimension refers to the presence or absence of symptoms and diagnoses associated with mental ill-health (e.g. anxiety disorders, depression). This dimension can be perceived as a spectrum, ranging from the absence of symptoms to the manifestation of mild to severe symptoms of mental ill-health. With both dimensions in mind, when alluding to mental health, this review will subsequently employ Keyes’ holistic conceptualisation, and refer to mental health as a complete state determined by the presence and absence of both *wellbeing* and *mental ill-health* [32, 33]. It is emphasised that a coach can therefore experience high levels of wellbeing and flourish within their profession (e.g. career progression and strong social connections) despite the diagnosis of a mental disorder (e.g. generalised anxiety disorder) [34].

In the extant literature, several reviews have explored the psychological health and experiences of coaches. These studies have summarised key concepts around stressors [21, 22, 35, 36], wellbeing [21, 35, 36], coping strategies [3, 21, 36] and burnout [37]. In adding to these, this review provides a unique overview of coach mental health by addressing current research gaps in four distinct ways. This is achieved by exploring mental health using an empirically supported framework, assessing elite-level coaches exclusively, exploring the risk and protective factors that influence coach mental health, and examining the relationship between mental health and coaching effectiveness.

Given previous reviews have failed to adopt a mental health framework to contextualise findings, the present review seeks to employ Keyes’ empirical framework to uniquely investigate the mental health of elite-level coaches. The model sets the review apart, as novel insights associated with the presence and prevalence of clinical symptoms and disorders in elite-level coaches will be considered concurrently with experiences of mental wellbeing for the first time. In adding to this, previous reviews have also largely examined coach mental health across various competitive levels, including those operating in both community and elite sports environments [21, 22, 36, 37]. In line with Olsen et al.’s [3] systematic review investigating coping among elite-level coaches, the present review will solely examine the mental health of elite-level coaches, to refine and focus the findings to a specific subset of the coaching population.

Beyond the exploration of mental health outcomes, the review will also seek to examine the various risk and protective factors that shape and influence the mental health of elite-level coaches. Purcell et al. propose that an ecological systems approach is beneficial to assess the various influences that impact the mental health of an elite athlete [38]. This socioecological approach asserts that an athlete’s mental health is influenced by factors that operate at the individual, microsystem (interpersonal), exosystem (organisational) and macrosystem (societal) level. Previous studies have employed this ecological approach to assess the risk and protective factors that contribute to the mental health of elite sportspeople [39]. Given the value of this socioecological approach, this review will seek to identify the various influences that operate as risk and protective factors for the mental health of elite-level coaches.

Finally, the present review seeks to examine the relationship between mental health (i.e. both wellbeing and mental ill-health) and coaching effectiveness. Often shaped by varying subjective and contextual objectives, coaching effectiveness is a term that has been expressed in multiple forms. The evaluation of performance for example, can fluctuate between intrapersonal and interpersonal viewpoints, as well as continuous (e.g. times and distance) and ordinal (e.g. ranking or league position) metrics [40]. Previous research has largely operationalised coaching effectiveness by employing Côté and Gilbert’s [41] proposed definition, “the consistent application of integrated professional, interpersonal and intrapersonal knowledge to improve athletes’ competence, confidence, connection, and character in specific coaching contexts” (Côté and Gilbert (2009, p.316). Recently however, Lyle [42] has suggested that coaching effectiveness should be considered as a superordinate concept as opposed to a root definition. Lyle argues that effectiveness should refer to the application of a coach’s expertise with the resources made available, rather than the satisfaction or achievement of certain criteria, whether that be process (e.g. improving athlete’s confidence) or outcome-oriented (e.g. performance output) objectives. This conceptualisation refrains from confining effectiveness to the achievement or satisfaction of a particular  set of standardised criteria (as put forward by Côté and Gilbert), but rather shifts the focus towards a broad and non-exhaustive range of factors that may influence the satisfaction of specific and relative goals and objectives set by a coach or sports organisation. These objectives may encompass targets associated with coaching practices, coach development and even performance-related outcomes. Ultimately, this perspective implies that effectiveness should be attainable for all coaches, and that excellence should not solely be confused with or utilised as a proxy for effectiveness.

Considering the importance of performing effectively, there may be an opportunity to utilise coaching effectiveness as a ‘hook’ to engage elite-level coaches in greater consideration of their mental health needs. It has been proposed that future research should examine coaching effectiveness from a wellbeing perspective [43], since preliminary evidence indicates that mental health may contribute positively and negatively towards factors such as productivity and motivation [13, 44–46]. Given elite-level coaches generally experience time-related challenges due to internally and externally imposed pressures [1, 4, 8], coaches may not consider the management of their own mental health as a competitive priority when compared with other influences (e.g. tactical advantages) [47]. Exploring the link between mental health and coaching effectiveness could therefore have potential value from a performance and ecological perspective. Coaches with a vested interest in mental health may subsequently enhance both their own psychological wellbeing and coaching effectiveness, as well as the mental health of individuals and communities who operate within their interpersonal environment (e.g. athletes and support staff).

Considering the features discussed, the present review aims to discern: (1) elite-level coaches’ experiences of mental wellbeing; (2) the nature and prevalence of mental ill-health in elite-level coaches; (3) the risk and protective factors that influence coach mental health; (4) how coaching effectiveness is conceptualised from a mental health perspective; and (5) the relationship between mental health and coaching effectiveness.

## Methods

A systematic scoping review methodology (as opposed to a systematic review) was employed due to the nascence of the field [48]. The study protocol was pre-registered via the Open Science Framework platform on the 9th of December 2021 (https://osf.io/zm63q/). Given the broad and emerging nature of the current discourse, this review was undertaken to gauge what is currently known about the mental health of elite-level coaches, and identify gaps that should be addressed by future research [48]. This provided an opportunity to generate a broad set of research questions aimed at mapping out the current evidence associated with elite coach mental health [49]. The study implemented a scoping review framework developed by the Joanna Briggs Institute (JBI) [50], and was consistent with the Preferred Reporting Items for Systematic reviews and Meta-Analyses extension for Scoping Reviews (PRISMA-ScR) guidelines [51] (see Additional file 1). Evidence-based and field-specific scoping review guidelines proposed by Sabiston et al. [49] were also considered, including the registration of a study protocol and the performance of a quality appraisal.

### Eligibility Criteria

The inclusion and exclusion criteria were established using the Population-Concept-Context (PCC) framework developed by the JBI Scoping Review Methodology Group [50, 52]. The population was classified as elite-level coaches who operate as part of the management team or leadership group, and regularly work with athletes or teams competing at the Olympic, Paralympic, international, national, professional or National Collegiate Athletic Association (NCAA) Division I level [53]. Although the term ‘elite’ has historically evaded a conceptually unified definition throughout sport, this research aligns with the International Olympic Committee (IOC) [54] and Reardon et al.’s [55] conceptualisation of elite sport. This definition was employed since previous characterisations and taxonomies tailored for elite athletes could not be directly translated or applied to elite-level coaches [56]. In addition, previous definitions of elite-level coaches have also failed to incorporate individuals operating at collegiate levels [57]. Given a number of previous studies have advocated for the inclusion of U.S. collegiate competitions as part of the elite sports domain [16, 56, 58, 59], this review sought to incorporate NCAA Division I coaches, but excluded those operating within Division II or III due to the regional emphasis of these competitions [60, 61]. Although this study’s conceptualisation may vary with other research, it has been argued that conceptual transparency is key, given the challenges of achieving a unified consensus due to the multi-faceted nature of elite sport (e.g. training time, individual versus team sport and professionalism) [62]. Coaches were also required to operate as part of a management or operational team. This criterion subsequently excluded backroom or support staff (e.g., sports psychologists and physiotherapists), as well as coaches whose primary responsibility involves some form of medical practice (e.g., strength and conditioning coaches). Both academy and retired coaches were also included within the review, as these individuals can offer prospective and retrospective insights respectively. Research that included heterogeneous samples (e.g. athletes, support staff, retired/academy coaches, or non-elite coaches) was only considered if they reported findings for elite-level coaches exclusively. Studies that failed to meet this criteria were subsequently excluded.

Conceptually, the present review employed Keyes’ [28, 29] dual-continua model of mental health to guide the classification of results, given this framework has been endorsed in an elite sports context [34, 63, 64] and empirically validated across a range of studies in the general population [32, 65, 66]. Findings associated with wellbeing were determined by various indices relating to an elite coach’s emotional, psychological and social wellbeing (e.g. quality of one’s life, positive functioning). Conversely, findings affiliated with mental ill-health were distinguished by criteria outlined in the Diagnostic and Statistical Manual of Mental Disorders (DSM-5; American Psychiatric Association, 2013), including symptoms associated with anxiety disorders, depression, post-traumatic stress disorders and substance use disorders for example. Psychological indicators and syndromes perpetuating (and often underlying) mental ill-health were also considered as part of this classification (e.g. burnout and distress). Despite the fact that indicators such as burnout are not typically considered to represent a mental disorder, there is considerable overlap between symptoms of burnout and depression [68]. Given this review’s broad and exploratory approach to mapping out the current body of evidence regarding elite coach mental health, it was deemed premature to exclude findings connected to burnout.

Although evidence associated with coaching effectiveness was not a prerequisite to satisfy the inclusion criteria, this review operationalised coaching effectiveness using Lyle’s [42] superordinate conceptualisation. Rather than adopting a root definition that may focus on meeting certain criteria or characteristics (e.g. performance outcomes or athlete connection) associated with coaching effectiveness, in line with the review’s expansive and investigative approach, this study employed a broad conceptualisation that referred to coaches’ drawing upon their expertise in the context of their specific ambition and environment. This conceptualisation focuses on potential factors and processes that may influence coaching gains or performance outcomes, but ultimately may not yield results and outcomes associated with success or excellence. These factors may encompass but are not constrained to various elements (e.g. resources, strategies, reasoning, actions) associated with coaching practices, coach education and coach development.

With regards to context, although elite-level coaches from specific demographics, sports or environments were not considered, the review required research to have been published in English and either during or after the year 2000. The decision to incorporate English-only studies was made as English was the first language of all authors. Studies published post-2000 were also selected exclusively in the attempt to incorporate and synthesise the most up-to date evidence. Other required details include being peer-reviewed, providing primary data, and having access to the full-text. The complete inclusion criteria can be observed in Table 1.

**Table 1:** Inclusion criteria

(1) Published in a peer-reviewed journal
(2) Published after January 2000
(3) Conducted primary research
(4) Published in English
(5) The full-text version was available (and if unavailable contact with the lead author was made, and if no response was received within two weeks the study was excluded)
(6) The study collected data from elite or high-performance coaches who manage athletes at the Olympic, Paralympic, national, international, professional or NCAA Division I level
(7) The study explored coaches who operate in leadership or management positions exclusively (e.g. head/assistant/senior coaches) or provide group findings separately where a heterogeneous sample (e.g. strength & conditioning coaches) is utilised
(8) The study explored coaches exclusively or provided group findings separately where a sample beyond coaches (e.g. athletes or support staff) was utilised
(9) The study explored elite coaches exclusively or provided group findings separately where a heterogeneous sample (e.g. elite and non-elite) was utilised
(10) The study reported on the mental health (mental wellbeing or mental ill-health) of elite-level coaches

### Search Strategy

A systematic search for peer-reviewed studies was undertaken at the end of December 2021 (Professional abstracts were also included providing the inclusion criteria was satisfied and results were interpretable.) Five electronic databases were selected, including: PsycINFO, MEDLINE, CINAHL, SPORTDiscus and Scopus. A university librarian was initially consulted to assist with developing keywords and search terms: “(‘Elite’ OR ‘High-performance’ OR ‘Olympic’) AND (‘Mental disorders’ OR ‘Mental health’ OR ‘Wellbeing’) AND (‘Coach’ OR ‘Manager’ OR ‘Director’)” (see Additional file 2 for the full search string). To ensure key studies were identified, a limited pilot search was conducted using PsycINFO, CINAHL and SPORTDiscus. Following the pilot search, the search strategy was modified to exclude terms associated with sport, athletes or specific sporting codes, as the search string was failing to detect key studies. In conjunction with the five electronic databases, Google Scholar was also utilised as a supplementary database, with the first 200 listings being examined to ensure all relevant studies had been identified [69].

Once all searches had been complete, each list was imported into Covidence review software and duplicates were initially removed [70]. The first author (JF) completed the title and abstract screening of all records. An additional reviewer (KF) independently screened 10% of records from a random sample to calibrate results. Where disagreements arose between authors, a discussion on whether a study should undergo full-text screening was settled between reviewers. Once the entire set of records had been examined at the title and abstract level, the first author (JF) engaged in full-text screening. A second author was consulted (CW) where the first author was uncertain about the eligibility of studies (16 studies). Following data charting, the first author (JF) conducted backward snowballing to ensure all relevant records had been identified within the included studies [71]. A secondary systematic search was also conducted in September 2022 to ensure newly published studies were incorporated within the review.

### Data Charting

After full-text screening was complete, information was charted using a data extraction tool adapted from Willis et al. [72] and the JBI’s extraction template [73] (see Additional file 3). Data extracted included information and findings associated with the author, study design, study objectives, participant details, conceptual frameworks, data analysis and results concerning the review’s research questions, amongst others. The tool was initially piloted on five relevant studies, where modifications were made to ensure key criteria were incorporated within the extraction form. Following this, each study that satisfied the full-text screening process underwent data charting by the first author (JF). Additionally, a secondary set of reviewers (KG and MK) independently charted data from 50% of the total studies each. After double extraction was completed, findings were inserted into a spreadsheet. A narrative synthesis was then undertaken to summarise and describe the current evidence base [74].

### Quality Appraisal

In line with recommendations put forward by Sabiston et al. [49], a quality appraisal was performed to critically examine the methodological rigour of studies that met the inclusion criteria. While quality assessments are not mandatory for conducting scoping reviews, quality appraisals offer transparency to the review process and help to contextualise findings that emerge from the included studies [49]. As a result, this review employed Hong et al.’s [75] Mixed Methods Appraisal Tool (MMAT), as the instrument provides reviewers with a single tool to assess quantitative, qualitative and mixed methods research, and has shown improved content validity over recent years [76]. The MMAT assesses five varying methodological criteria contingent upon the study design selected [75]. For this review, each study was initially categorised into either qualitative, randomised controlled, nonrandomised, quantitative descriptive or mixed method research designs. Once classified, a reviewer (JF) assessed whether the selected study had met the requirements to satisfy the proposed criteria. Parameters were interpreted and determined using Hong et al.’s proposed indicators and explanations to assess the relevant criteria (see the MMAT’s user guide for further clarification [77]). The reviewer then judged each criterion by providing a ‘Yes’, ‘No’ or ‘Can’t Tell’ response to evaluate whether the criteria had been satisfied. Once complete, to assess the methodological robustness of each study, the ‘Yes’ responses were tallied up and converted into percentages to examine the proportion of criteria that had been satisfied.

## Results

**Fig. 1:**
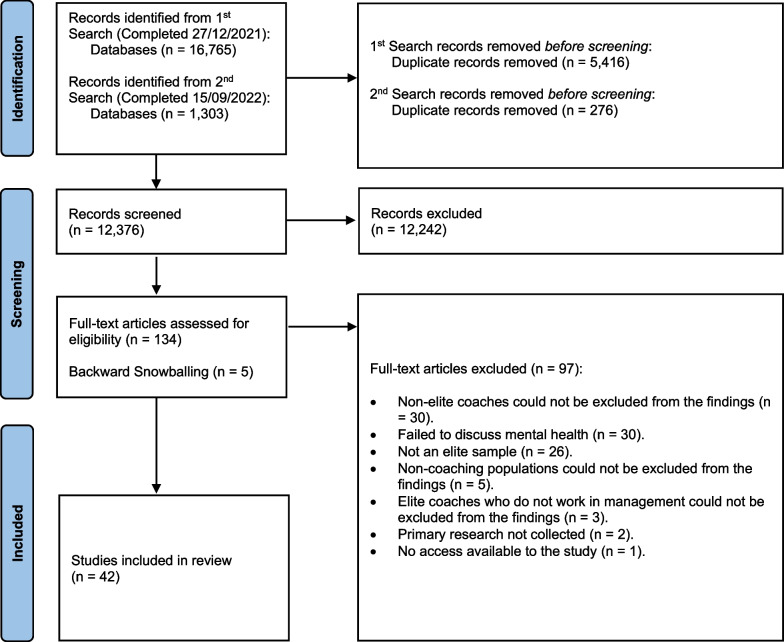
PRISMA-ScR flow diagram

The systematic search yielded a total of 18,068 records, of which, 5,692 were identified as duplicates (Fig. 1). As a result, 12,376 records were screened at the title and abstract level, before 134 studies were deemed eligible for full-text screening. From there, 97 studies failed to satisfy the overall inclusion criteria, leaving a total of 42 studies eligible for data charting (5 studies were identified from backward snowballing). Of the included studies, 5 were classified as either a professional abstract [78–81] or editorial letter [82].

### Study Characteristics

Of the 42 included studies, 29 were quantitative (69.1%), 10 qualitative (23.8%) and 3 adopted a mixed-methods approach (7.1%). Nineteen studies were cross-sectional (45.2%), 11 longitudinal (26.2%), 4 employed a case study methodology (9.5%) and 3 were experimental (7.1%). The quality appraisal revealed that 6 studies met 100% of criteria (14.3%), whilst 3 met 80% (7.1%), 8 met 60% (19%), 16 met 40% (38.1%), 7 met 20% (16.7%) and 2 met 0% (4.8%) of criteria (see Additional file 4).

Across the 42 studies there were 4,576 participants in total (Table 2). Twenty-one studies included a mixed sex sample (50%), 13 explored males exclusively (31%), 2 reported on females exclusively (4.7%), and 6 failed to specify sex or gender (14.3%).^1^ Of the 4,576 participants, 3,325 were male (72.7%), 821 were female (17.9%) and 1 was gender diverse (< 0.1%). Coaches ranged from 18 to 74 years of age, and most studies possessed a mean age range between 35 to 44 (*n* = 16, 38.1%).  Europe served as the primary setting for most studies (*n* = 27, 64.3%), followed by Oceania (*n* = 8, 19%) and North America (*n* = 5, 11.9%). Two studies were also conducted in Asia (*n* = 2, 4.8%) and 1 was carried out in South America (*n* = 1, 2.4%).

**Table 2:** Summary of included studies

Study	Study Design; MMAT Quality Score	N (male: female)	Themes	Sport; country	Definition of elite	Key findings
Åkesdotter et al. [108]	Quantitative, Cross-sectional; 40%	34 (23:11)	Alcohol-Related Disorders Anxiety, Depression, Eating Disorders, Substance-Related Disorders	Various; Sweden	International	Of the 34-treatment seeking high-performance coaches, 93% were diagnosed with any anxiety disorder, 28% were diagnosed with a major depressive disorder (single episode or recurrent), and 14% were diagnosed with an alcohol-related disorder. Less than 5 coaches were diagnosed with either an eating, bipolar, or substance-related disorder
Baldock et al. [10]	Qualitative; 100%	8 (8:0)	Wellbeing	Soccer; United Kingdom	National, Professional	Coaches largely appraised performance, organisational and personal-related stressors as threats. These demands were predominantly met with ineffective coping strategies, which subsequently led to negative states of wellbeing. Conversely, when effective coping strategies were employed, coaches usually experienced high levels of wellbeing
Baldock et al. [83]	Mixed-Methods, Longitudinal; 80%	18 (18:0)	Burnout, Wellbeing	Soccer; United Kingdom	National, Professional	Using four time points across a competitive season, coaches reported moderate levels of wellbeing and low to medium levels of burnout. Wellbeing was at its lowest at the beginning of the season, whilst emotional exhaustion and depersonalisation were highest at the end of the season
Balk et al. [90]	Quantitative, Longitudinal; 60%	31 (30:1)	Wellbeing	Various; Australia and The Netherlands	International, National	Emotional detachment (the previous day), sleep quality and sleep duration contributed towards a coach’s experience of positive affect the following morning. Coaches who reported greater positive affect were also more likely to experience high levels of work engagement
Bentzen et al. [97]	Qualitative; 40%	4 (4:0)	Burnout	Not Specified; Denmark and Norway	Professional	Organisational and performance-related stressors contributed towards a shift in the quality of motivation maintained by a coach. A shift from autonomous to controlled motivation operated as an antecedent to burnout. Coaches also experienced a range of symptoms associated with burnout, including affective, cognitive and behavioural changes
Bentzen et al. [96]	Quantitative, Longitudinal; 40%	299 (274:25)	Burnout (Emotional Exhaustion)	Various; Norway and Sweden	National	A significant increase in emotional exhaustion was reported by elite-level coaches over the course of a season. Although the majority of the sample reported low levels of exhaustion throughout the season (71%), 24.4% of coaches met the criteria for high levels of exhaustion. High workload and work-home interference were associated with high levels of exhaustion, and high levels of recovery, intrinsic regulations and identified regulations were associated with low levels of emotional exhaustion
Bentzen et al. [87]	Quantitative, Longitudinal; 40%	343 (313:30)	Burnout, Wellbeing	Various; Norway and Sweden	National	A small to moderate increase in burnout and decrease in wellbeing occurred in elite-level coaches over the course of a competition season. No significant relationship was identified between burnout and season length, thus suggesting that the length of a season may not be a contributing factor towards burnout
Bentzen et al. [88]	Quantitative, Longitudinal; 40%	299 (274:25)	Burnout, Wellbeing	Various; Norway and Sweden	International, National	Coaches who reported high levels of job insecurity in the middle of the season were more likely to experience low levels of mental wellbeing and high levels of exhaustion and cynicism at the end of the season. Job value incongruence did not significantly impact levels of wellbeing and burnout over the season
Bentzen et al. [24]	Qualitative; 100%	6 (5:1)	Wellbeing	Soccer; Norway	National	Coaches experienced a decrease in occupational wellbeing when fired. They also experienced a range of negative (e.g. frustration) and positive (e.g. gratitude) emotional responses throughout the process
Carling et al. [82]	Quantitative, Experimental; 20%	6 (N/A)	Anxiety, Sleep	Soccer; France	National	The developed music intervention helped significantly shorten the time required to fall sleep, as well as reduce symptoms of anxiety among elite-level coaches
Carson et al. [92]	Quantitative, Cross-sectional; 40%	128 Elite (464 Overall) (N/A)	Wellbeing	Not specified; Australia	International, National	Australian high-performance coaches generally reported high levels of wellbeing on the Warwick-Edinburgh Mental Wellbeing Scale (M = 51.7, SD = 8.3)
de Sousa Pinheiro et al. [94]	Quantitative, Longitudinal; 40%	26 (26:0)	Wellbeing	Soccer; Brazil	National	Coaches reported significantly higher levels of stress in competition and during training periods when compared with vacation periods. No significant differences in stress levels were observed between competition and training periods. With regards to recovery, results were only significantly higher in vacation periods compared to competitive periods
Foretić et al. [109]	Quantitative, Case Study; 40%	1 (N/A)	Anxiety	Handball; Qatar	National, Professional	Coaches experienced significantly greater post-match anxiety when compared with anxiety levels post-training. These findings were physiologically corroborated with biomarkers, as cortisol, alpha-amylase and heart rate levels were significantly higher prior to matches as opposed to training. These findings demonstrate evidence of a ‘pre-competitive’ effect amongst elite-level coaches
Gencay and Gencay [101]	Quantitative, Cross-sectional; 40%	65 (55:10)	Burnout	Judo; Turkey	National, Professional	Elite-level judo coaches experienced medium levels of burnout on average. No significant associations were identified between gender; however, a positive correlation was discovered between emotional exhaustion and years of coaching experience. Coaches were also more likely to be protected from burnout if they perceived themselves as being supported by their sports administrators
Georgios and Nikolaos [102]	Quantitative, Cross-sectional; 20%	164 (164:0)	Burnout	Track & Field; Greece	National	Hardiness, competitive trait anxiety and satisfaction with support all negatively predicted a coach’s perception of stress, whilst hardiness and satisfaction with support negatively predicted burnout. Competitive trait anxiety and perceived stress also positively predicted burnout
Hägglund et al. [84]	Qualitative, Experimental, Longitudinal; 60%	18 (7:11)	Wellbeing	Athletics and Figure Skating; Sweden	International, National	The mindful self-reflection intervention contributed towards an increase in practices associated with wellbeing (e.g. self-compassion) amongst elite-level coaches. These behaviours were still being implemented at 6- and 12-month follow-ups post-intervention
Hassmén et al. [98]	Mixed-Methods, Longitudinal; 20%	8 (8:0)	Burnout	Soccer; Sweden	National	Coaches experienced fluctuating levels of emotional exhaustion and depersonalisation over the 10-year study period. Withdrawal from the profession led to significant reductions in emotional exhaustion
Hjälm et al. [99]	Quantitative, Cross-sectional; 80%	47 (47:0)	Burnout	Soccer; Sweden	National, Professional	71% of coaches managing soccer teams from the Women’s Premier League in Sweden reported moderate to high levels of emotional exhaustion. Conversely, only 23% and 45% of coaches managing teams within the Swedish Men’s Premier League and Men’s Second League reported moderate to high levels of emotional exhaustion respectively
Kaski and Kinnunen [93]	Quantitative, Cross-sectional; 40%	499 (384:115)	Burnout; Wellbeing	Not Specified; Finland	International, National	Although Finnish coaches generally experienced good levels of wellbeing (work engagement), 22% of coaches met the criteria for mild symptoms of burnout, and 2% met the criteria for severe symptoms of burnout. A lack of job resources was found to predict burnout and work engagement more effectively than the presence of job demands
Kegelaers et al. [11]	Quantitative, Cross-sectional; 40%	119 (97:22)	Anxiety, Depression, Risky Alcohol Consumption, Sleep, Psychological Distress	Not Specified; Belgium and The Netherlands	International, National	39.5% of the coaching sample reported symptoms associated with depression or anxiety, and 19.3% indicated experiences of distress. 19.3% of coaches met the caseness criteria for adverse alcohol use and 25.2% reported sleep disturbance
Kellmann et al. [95]	Quantitative, Longitudinal; 60%	6 (6:0)	Wellbeing	Australian Football; Australia	National, Professional	On average, coaches maintained low to moderate levels of stress over the course of a season, whilst recovery levels were low to moderate and declined throughout the season. Vacations also seemed to contribute to a temporary recovery effect for coaches. Sleep quality however was consistently low throughout the season
Kenttä et al. [86]	Qualitative, Case-Study; 100%	1 (0:1)	Wellbeing	Swimming; Sweden	International, Olympic	The coaches relieved from their duties experienced compromised mental wellbeing (cognitive and emotional) including symptoms associated with anxiety and distress post-termination. Effective psychological skills and strong social supports were perceived as important protective factors during this process
Kenttä et al. [85]	Qualitative; 60%	37 (0:37)	Wellbeing	Various; Sweden	National	Although female coaches largely experienced the same challenges as male coaches, a unique set of sex-specific stressors emerged from the study, including proving oneself to male counterparts, lack of acceptance in the coaching role, and receiving less support at home due to family-related responsibilities. Participants revealed adapting to the stereotypical male culture of sport in order to protect their mental wellbeing, as opposed to challenging cultural norms
Kim et al. [111]	Quantitative, Cross-sectional; 60%	69 (53:15 – 1 gender diverse)	Depression	Soccer and Various; New Zealand	International, National	14.1% of coaches met the criteria for at least moderate symptoms of depression. Contemplating retirement and having a family history of a mood disorder was significantly associated with symptoms of moderate or major depression. A strong association was also discerned between the total and severity of daily life hassles and symptoms associated with depression
Lee and Chelladurai [89]	Quantitative, Cross-sectional; 40%	430 (278:152)	Burnout (Emotional Exhaustion), Wellbeing	Not Specified; U.S	NCAA Division I	Positive affectivity predicted 3 forms of emotional labour (surface acting, deep acting, genuine expression), whilst negative affectivity predicted surface acting amongst coaches. Positive affectivity, negative affectivity, surface acting, and genuine expression all predicted emotional exhaustion too. The relationship between surface acting and emotional exhaustion was also significantly moderated by emotional intelligence
Lee [103]	Quantitative, Cross-sectional; 60%	203 (115:88)	Anxiety, Burnout (Emotional Exhaustion)	Various; U.S	NCAA Division I	Goal incongruence positively predicted and coping efficacy negatively predicted subjective ratings of anxiety amongst NCAA Division I coaches. In addition, self-reported levels of anxiety also positively predicted self-reported emotional exhaustion
Longshore and Sachs [91]	Mixed-Methods, Experimental; 20%	20 (8:12)	Anxiety, Wellbeing	Not Specified; U.S	NCAA Division I	Coaches experienced significantly less positive affect, negative affect and trait anxiety following the Mindfulness Training for Coaches Program. Greater emotional stability contributed towards enhanced wellbeing and coach-athlete interactions
Lundkvist et al. [100]	Qualitative; 100%	8 (8:0)	Burnout	Soccer; Sweden	National	Two profiles of burnout were identified in elite-level coaches. Those who burned out due to the performance culture of elite sport experienced behavioural changes (e.g. passive and asocial). Conversely, coaches who experienced considerable work-home challenges (i.e. life-situation profile) experienced high levels of exhaustion (e.g. fatigue and mental overload)
Lundkvist et al. [104]	Quantitative, Cross-sectional; 60%	97 Elite (336 Overall) (N/A)	Burnout (Emotional Exhaustion)	Soccer; Sweden	International, National, Professional	In alignment with MBI and clinical cut-offs, this cohort of coaches presented low levels of emotional exhaustion in comparison to normative samples
Nikolaos [105]	Quantitative, Cross-sectional; 20%	170 (170:0)	Burnout	Basketball; Greece	National	On average, coaches experienced moderate levels of emotional exhaustion and depersonalisation, and low levels of personal accomplishment. Coaching level, social support and coaching issues significantly predicted the stress appraisal, whereas stress appraisal, social support and coaching issues significantly predicted burnout
Olusoga et al. [13]	Qualitative; 100%	12 (6:6)	Wellbeing	Various; United Kingdom	International, Olympic	Coaches revealed that perceived stress contributed towards negative affective states, including feelings associated with emotional fatigue and depression
Olusoga and Kenttä [45]	Qualitative, Case Study; 80%	2 (2:0)	Burnout	Not Specified, Sweden	National, Olympic	Burnout generally occurred due to a lack of recovery time for coaches, as opposed to the overload of performance, organisational and personal-related stressors. Departing the coaching role and possessing adequate social support were key factors in recovering from burnout
Pilkington et al. [12]	Quantitative, Cross-sectional; 40%	78 Coaches (252 including High-Performance Support Staff) (59:19)	Anxiety, Depression, Risky Alcohol Consumption, Sleep, Psychological Distress, Wellbeing	Not Specified; Australia	International, National	43.6% of coaches met the probable caseness criteria for anxiety and depression, whereas 48.1% and 10.3% met the caseness criteria for risky alcohol consumption and psychological distress respectively. 34.6% of coaches also revealed they had sought psychological treatment at some stage throughout their life
Roberts et al. [110]	Qualitative, Case Study; 100%	1 (1:0)	Depression, Risky Alcohol Consumption	Not Specified, United Kingdom	International	The head coach described concealing their experiences of depression and alcoholism due to the stigma associated with mental ill-health in elite sport. The coach also revealed a desire to conceal their mental health status in order to ensure future employment was a possibility
Ruddock et al. [78]	Quantitative, Cross-sectional; 0%	142 (N/A)	Anxiety, Burnout, Depression, Psychological Distress	Australian Football; Australia	National, Professional	Findings revealed that emotional exhaustion was the only component of burnout that could predict levels of anxiety, depression and stress amongst professional AFL (Australian Football League) coaches. Greater levels of emotional exhaustion contributed towards higher levels of anxiety, depression and stress
Ruddock et al. [79]	Quantitative, Longitudinal; 20%	115 (115:0)	Anxiety, Burnout, Depression, Psychological Distress	Australian Football, Australia	National, Professional	Emotional exhaustion, stress and overall DASS and GHQ scores significantly increased from pre-season to mid-season. Emotional exhaustion was also identified as the strongest predictor of mental ill-health, as high levels of stress, anxiety and depression were all significantly predicted by emotional exhaustion
Ruddock et al. [80]	Quantitative, Longitudinal; 20%	50 (N/A)	Anxiety, Burnout, Depression, Psychological Distress	Australian Football; Australia	National, Professional	Concomitant increases of emotional exhaustion, depression and stress occurred between February and August when contract renewal was approaching. These levels started to decrease in November once the competitive season had concluded
Ryska [106]	Quantitative, Cross-sectional; 40%	345 (267:78)	Burnout	Various; U.S	NCAA Division I	Coaches generally reported low levels of burnout. Those who pursued prestige goals and public relations using a high strategic-low collaborative relationship were more likely to experience burnout, whereas those who pursued personal growth and athletic excellence with a low bureaucratic-high collaborative leadership were less likely to suffer burnout
Seo et al. [107]	Quantitative, Cross-sectional; 60%	210 (151:59)	Burnout	Taekwondo; South Korea	National	Workplace conditions negatively predicted burnout, whereas burnout positively predicted presenteeism in South Korean National coaches. Those operating within a positive working environment were less likely to suffer burnout and presenteeism
Smith et al. [81]	Quantitative, Cross-sectional; 0%	21 (7:14)	Eating Disorders	Cheerleading; U.S	NCAA Division I	38.1% of coaches were considered to be at risk of an eating disorder. Pathogenic behaviours included risk of using laxatives, diet pills or diuretics (33.3%), loss of 20 or more pounds in a month (9.5%), risk of vomiting (9.5%) and risk of binge eating (4.8%)
Smith et al. [113]	Quantitative, Cross-sectional; 40%	35 Elite (202 Overall) (25:10)	Various Mental Illnesses	Various; United Kingdom	Olympic, Paralympic	45.7% of high-performance coaches from the sample had experienced or were currently experiencing a mental disorder
Vinberg et al. [112]	Quantitative, Cross-sectional; 40%	401 Coaches (317:79–5 missing)	Gambling Disorders	Various; Sweden	National, Professional	6% of coaches demonstrated at-risk gambling behaviours

When identifying the type of sports that participants coached, 18 (42.9%) studies assessed elite-level coaches who managed team sports exclusively. Nine (21.4%) studies reported coaches managing athletes from both individual and team sports, and only one study explored a coach managing an individual sport (2.4%). Frequently cited sporting codes included soccer/football (*n* = 17, 30.4%), swimming (*n* = 7, 12.5%) and basketball (*n* = 9, 10.7%). Regarding the ‘elite’ criteria, 31 (73.8%) included studies examined coaches operating at the national level. This was followed by international (*n* = 13, 31%), professional (*n* = 12, 28.6%), NCAA Division I (*n* = 5, 11.9%), Olympic (*n* = 4, 9.5%) and Paralympic (*n* = 1, 2.4%) samples.

### What are Elite-Level Coaches’ Experiences of Wellbeing?

This review found that 17 (40.5%) studies explored wellbeing in elite-level coaches. Seven of these studies investigated wellbeing using semi-structured interviews [10, 13, 24, 83–86]. A number of measures were also utilised to explore the various dimensions of wellbeing, including the Satisfaction with Life Scale (SWLS; *n* = 3) [12, 87, 88], Positive and Negative Affect Schedule (PANAS, multiple versions; *n* = 3) [89–91], Warwick-Edinburgh Mental Well-being Scale (WEMWBS; *n* = 2) [83, 92], Subjective Vitality Scale (SVS; *n* = 2) [87, 88], Utrecht Work Engagement Scale (UWES; *n* = 2) [90, 93], Recovery-Stress-Questionnaire (RESTQ, multiple versions; *n* = 2) [94, 95] and Brunel Mood Scale (*n* = 1) [91]. The findings indicate that elite-level coaches generally experience moderate to high levels of mental wellbeing [83]. Carson et al. for instance [92] found that elite-level coaches from Australia reported high levels of mental wellbeing on the WEMWBS. This outcome was supported by Kaski and Kinnunen [93], where a sample of elite-level Finnish coaches generally reported good levels of positive functioning on the UWES.

Amongst correlates and predictors of wellbeing, in comparison to performance and personal-related stressors, preliminary evidence indicates that demands emanating from organisational sources may contribute most significantly towards coaches’ experience of low hedonic and eudaimonic wellbeing [10]. Despite the proliferation of stressors, Baldock et al. found that coping effectiveness was perceived to have the largest influence on a coach’s experience of mental wellbeing. Other reported correlates and antecedents of high wellbeing include psychological detachment from coaching [90], adequate sleep quality and duration [90], autonomy [96], autonomy support [96] and relatedness [96]. Conversely, excessive workloads [87] and persistent job insecurity [24] were associated with low levels of wellbeing.

With these factors in mind, two experimental investigations examined whether levels of mental wellbeing could be improved amongst elite-level coaches. Longshore and Sachs found that coaches experienced significantly reduced negative affect after undertaking a 6-week mindfulness training program [91]. Given the small and nonrandomised sample however (*n* = 20), results should be interpreted with caution. Hägglund and colleagues found similar results when elite-level coaches were presented with a daily or weekly text message containing a mindful self-reflection [84]. Findings revealed that coaches experienced greater engagement with their mental wellbeing after the intervention (e.g. self-awareness, vulnerability and self-compassion), with lasting behaviours extending up to 12 months.

### What is the Nature and Prevalence of Mental Ill-Health in Elite-Level Coaches?

Thirty-two (76.2%) of the included studies examined themes associated with mental ill-health. Studies that satisfied the inclusion criteria explored symptoms associated with burnout (*n* = 21, 50%) [45, 78–80, 83, 87–89, 93, 96–107], anxiety (*n* = 10, 23.8%) [11, 12, 78–80, 82, 91, 103, 108, 109], depression (*n* = 8, 19%) [11, 12, 78–80, 108, 110, 111], psychological distress (*n* = 5, 11.9%) [11, 12, 78–80], risky alcohol consumption/disorders (*n* = 4, 9.5%) [11, 12, 108, 110], sleep disturbance/disorders (*n* = 3, 7.1%) [11, 12, 82], eating disorders (*n* = 2, 4.8%) [81, 108], substance abuse (*n* = 1, 2.4%) [108] and gambling disorders (*n* = 1, 2.4%) [112]. Nine studies (21.4%) [11, 12, 81, 93, 96, 99, 111–113] also examined the prevalence of mental ill-health amongst general elite-level coaching populations, and one study (2.4%) [108] explored the prevalence of psychiatric disorders in a treatment-seeking population.

#### Anxiety and Depression

Among the included studies, 10 examined symptoms associated with anxiety and 8 explored symptoms associated with depression. Symptoms of both anxiety and depression were measured using the General Health Questionnaire (GHQ, 12- and 28-item; *n* = 4) [11, 12, 78, 79] and Depression Anxiety Stress Scale (DASS-21; *n* = 3) [78–80], whilst the State-Trait Anxiety Inventory (STAI; *n* = 2) [91, 109] and Sport Emotion Questionnaire (SEQ; *n* = 1) [103] were employed to assess symptoms of anxiety, and the Center for Epidemiologic Studies Depression Scale—Revised (CESD-R; *n* = 1) [111] was utilised to examine symptoms of depression. Clinical interviews (*n* = 1) [108] and semi-structured interviews (*n* = 1) [110] were also employed to investigate symptoms of anxiety and depression.

Three studies examined the prevalence of anxiety and depressive symptoms in the broader elite-level coaching population. Kegelaers and colleagues reported that symptoms of depression and anxiety were reported by 39.5% of a Dutch and Flemish sample (*n* = 119) [11], aligning with the probable caseness percentages (43.6%) reported by 78 elite-level coaches from Australia [12]. In contrast, Kim and colleagues [111] found that only 14.1% of a New Zealand coaching sample (*n* = 69) met the cut-off criteria for at least moderate symptoms of depression. In addition to the broader elite-level coaching population, Åkesdotter and colleagues also found that 69% of a treatment-seeking coaching sample (*n* = 34) were experiencing clinical levels of anxiety, and 28% had met the diagnostic criteria for a major depressive disorder.

A number of correlates and antecedents associated with symptoms of anxiety and depression were identified within the included studies. For instance, both goal incongruence and coping efficacy were found to be significant predictors of self-reported anxiety [103], whereas contemplation of retirement, a family history of a mood disorder, and the frequency of daily hassles were identified as significant predictors of depressive symptoms [111]. One relationship of growing importance includes the association between burnout and anxiety/depression. It was found that emotional exhaustion, for example, predicted symptoms of anxiety and depression amongst two samples of professional Australian Rules coaches [78, 79].

#### Burnout

The included studies reported heterogeneous rates of burnout. The results ranged from low [83, 93, 96, 104–106], to moderate [83, 99, 101, 105], to high [99] levels of emotional exhaustion, depersonalisation and personal accomplishment. These findings were predominantly measured using a version of the Maslach Burnout Inventory (MBI), including a non-specified version (*n* = 9) [78–80, 89, 101, 102, 104–106], the MBI-General Scale (MBI-GS) (*n* = 4) [87, 88, 93, 96], MBI-Educators Survey (MBI-ES) (*n* = 2) [98, 99] and MBI-Coach (MBI-C) survey (*n* = 1) [83]. Other measurement tools included the Coach Burnout Questionnaire (CBQ) (*n* = 1) [103] and Coach Burnout Affect Survey (*n* = 1) [107]. In addition to quantitative measures, 5 studies also utilised semi-structured interviews to explore burnout [45, 83, 97, 98, 100].

When examining prevalence rates, Kaski and Kinnunen conducted a cross-sectional study and found that 22% and 2% of a Finnish coaching sample satisfied the criteria for mild to severe symptoms of burnout, respectively (*n* = 499) [93]. The results however also indicated that 76% of the coaching sample were not experiencing burnout. Another cross-sectional study carried out with ‘highly exhausted’ coaches at risk of burnout [97], found that a coach’s perception of their sports organisation (e.g. poor relations with upper management and leaders) and their everyday work environment (e.g. high workload and athlete win or loss records) was associated with the onset of burnout. Similarly, Hassmén and colleagues [98] explored the symptoms and recovery processes of elite-level coaches at risk of burnout over a period of ten years. They found that taking time off, departing the coaching profession, or stepping down to a lower competitive division was associated with lower levels of emotional exhaustion. By removing oneself from the coaching environment, participants were able to reduce their workload and gain greater autonomy, which subsequently lead to reduced symptoms of burnout.

#### Psychological Distress

Five of the included studies explored symptoms associated with psychological distress. Psychometric instruments included the DASS-21 (*n* = 3) [78–80], Kessler-10 (K-10, *n* = 1) [12] and Distress Screener (*n* = 1) [11]. Rates of high psychological distress ranged from 10.3% to 19.3% in elite-level coaches. A number of correlates were found to lower psychological distress, including satisfaction with life balance (i.e. wellbeing), satisfaction with social support and older age [12], whereas emotional exhaustion [78, 79] and depersonalisation [79] were correlated with high levels of psychological distress. Longitudinal research also found that professional Australian Rules Football coaches experienced significantly higher levels of psychological distress mid-season when compared to pre-season [79].

#### Sleep Disturbance

Two cross-sectional studies explored the prevalence of sleep disturbance amongst elite-level coaches, reporting comparable rates ranging from 23.4% (moderate to severe sleep disturbance) to 25.2% (presence of sleep disturbance) [11, 12]. These rates were identified using the Athlete Sleep Screening Questionnaire (ASSQ) and Patient Reported Outcomes Measurement Systems (PROMS) respectively. Findings also revealed that sleep disturbances were associated with symptoms of burnout [45, 97, 100], and that sleep quality has the potential influence a coach’s mental wellbeing (e.g. positive affect) [90]. There is some evidence to suggest however, that music could be utilised as a tool to assist elite-level coaches falling asleep [82].

#### Other Mental Disorders

A range of other symptoms associated with mental ill-health have also been explored among elite-level coaches. Two studies examined rates of risky alcohol consumption in elite-level coaches using the Alcohol Use Disorder Identification Tool-Concise (AUDIT-C), where the prevalence of symptoms ranged from 19.3% to 48.3% [11, 12]. Comparatively, Åkesdotter and colleagues also found that 17% of a treatment-seeking population met the clinical diagnosis for a psychoactive substance-use disorder (e.g. alcohol or substance-related disorder) [108]. Beyond substance use, Smith and colleagues identified 38.1% of a NCAA Division I coach sample (*n* = 21) being at risk of an eating disorder using the Eating Attitudes Test (EAT-26) [81]. Frequent pathogenic behaviours included risk of using laxatives, diet pills or diuretics (33.3%), loss of 20 or more pounds in a month (9.5%), risk of vomiting (9.5%) and risk of binge eating (4.8%). Furthermore, in a sample of professional coaches from Sweden (*n* = 401), it was reported that 6% met the criteria for risky gambling behaviours on the Problem Gambling Severity Index (PGSI) [112].

### What Risk and Protective Factors Contribute to Mental Health Amongst Elite-Level Coaches?

In line with the socioecological approach described by Purcell and colleagues [38], this review identified a variety of factors influencing coach mental health from within the broader ecology of elite sport (see Additional file 5). At the individual level, effective psychological skills or emotional regulation (e.g. hardiness and resilience) (*n* = 6), effective coping strategies (*n* = 5), exercise (*n* = 2), high levels of mental wellbeing (*n* = 2), coaching experience/age (*n* = 2), working full-time/part-time (*n* = 2), external non-coaching identities (*n* = 2), high intrinsic and identified motivations (*n* = 1) and collaborative leadership styles (*n* = 1) were considered protective. Conversely, risk factors included stress-related factors (*n* = 7), ineffective coping strategies (*n* = 3), mental ill-health comorbidity (e.g. symptoms of burnout) (*n* = 3), coaching experience/age (*n* = 3), low autonomous motivations (*n* = 2), maladaptive perfectionism (*n* = 2), gender (women) (*n* = 2), transitional phases (e.g. retirement) (*n* = 2), low levels of wellbeing (*n* = 1), low emotional intelligence (*n* = 1), controlling leadership styles (*n* = 1), working full-time (*n* = 1), family history of a mood disorder (*n* = 1) and a dominant coaching identity (*n* = 1). Beyond the individual, at the microsystem level, findings revealed that strong social support (e.g. partners, families, colleagues, athletes and mentors) (*n* = 8) and engaging with a psychologist (*n* = 2) operate as key protective mechanisms, whilst a lack of social support is considered a risk factor (*n* = 3).

From an exosystem perspective, protective factors were associated with the perceived working environment, including support provided by organisations and federations (*n* = 6), sufficient recovery (e.g. psychological and physical) (*n* = 2) and a reduced workload (*n* = 1). Similarly, risk factors were associated with negative perceptions of the working environment, including a high workload (*n* = 6), lack of organisation and federation support (*n* = 4), lack of recovery (e.g. psychological detachment or relaxation) (*n* = 4), poor work-life balance (*n* = 2), job insecurity (*n* = 2), excessive organisational interference (*n* = 1) and sport type (e.g. team sports) (*n* = 1). At the macrosystem level, no protective factors were identified, but stigma towards help-seeking (*n* = 2), the sporting culture (e.g. hypermasculinity, constant pressure to perform) (*n* = 2) and media scrutiny (*n* = 1) were identified as risk factors.

### What are the Various Ways Coaching Effectiveness is Conceptualised from a Mental Health Perspective, and How is Mental Health Associated with Coaching Effectiveness?

This review found preliminary evidence to suggest that a coach’s mental health may impact their ability to coach effectively. Results indicated that an elite coach’s mental health may influence either their own or athlete/team’s functioning (see Additional file 6). There was some support among the included studies to suggest that a coach’s mental health might impact their own psychological/emotional state, standard of work and coaching style. Psychological and emotional impacts ranged from changes in focus (*n* = 3), decision making (*n* = 1), emotional regulation (*n* = 1) and confidence (*n* = 1). For example, it was reported that elite-level coaches experiencing symptoms associated with anxiety may encounter challenges in pursuing short- and long-term goals [100, 103]. Furthermore, the findings also showed that a coach’s mental health may impact one’s standard of performance and coaching style. Several studies found that an elite coach’s mental health may influence their motivation (*n* = 1), presenteeism (*n* = 1) or work engagement (*n* = 1). For instance, it was found that burnout significantly increased presenteeism [107], and emotional exhaustion contributed towards a decrease in motivation amongst elite-level coaches [45]. Further analysis revealed that an elite-level coach’s mental health may impact their coaching style, including changes in verbal communication (*n* = 1) and leadership styles (*n* = 1). Lundkvist et al. reported that coaches who were experiencing burnout may adopt a passive style of leadership, where coaches tended to be quieter when addressing or interacting with their athletes [100].

In contrast, research assessing a coach’s impact upon their athlete or team’s functioning was scarce. The limited findings however, suggested that a coach’s mental health may influence an athlete/team’s standard of performance (*n* = 1). Olusoga and Kenttä found that elite-level coaches experiencing symptoms associated with burnout reported struggling to get the best out of their athletes [45].

## Discussion

This scoping review identified and synthesised peer-reviewed evidence exploring the mental health of elite-level coaches. The findings shed light upon the mental health outcomes of elite-level coaches, as well as the various factors that shape and influence levels of mental wellbeing and symptoms of mental ill-health. The review also sought to explore the relationship between mental health and coaching effectiveness, where findings can potentially be utilised to inform areas of further research, and supply elite-level coaches with preliminary evidence that may incentivise engagement with mental health awareness and practices. Overall, 42 studies were identified, with 40% and 76% of studies exploring themes associated with wellbeing and mental ill-health, respectively. Quality appraisal scores also suggest the field lacks sufficiently high-quality evidence to date. In particular, there is need to ensure that quantitative research is conducted with validated and reliable measures, and that researchers outline sampling strategies (e.g. snowball or purposive sampling) and sample size justifications (e.g. a priori power analyses) to ensure transparent and valid reporting.

Given 72% of the overall sample were male, it is important to acknowledge that the results may not generalise to other sexes or genders. The over-representation of male coaches in the current evidence base conforms to the broader sex/gender representation and imbalance that prevails throughout the field of sports psychology [114]. Although this underrepresentation may reflect the true proportion of coaches that identify as female in elite sport [115], more research is required to understand how female and non-binary coaches experience mental health to assist with targeted interventions. It is worth acknowledging however, that the proportion of male and female elite-level coaches may vary between sporting codes and elite settings. Certain sports (e.g. gymnastics) and specific elite environments (e.g. NCAA Division I) for instance, may feature a more balanced representation between male and female coaches, while others might be dominated by female coaches.

From a wellbeing perspective, the results revealed that elite-level coaches generally reported moderate to high levels of mental wellbeing. A number of individual-level factors were found to improve an elite coach’s wellbeing, including robust psychological skills, effective coping strategies, strong intrinsic and identified regulations, experience as a coach and regular exercise. Although individual-level factors such as self-compassion and self-awareness can be altered and improved to promote the wellbeing of elite-level coaches [84, 91], it should be noted that organisational and societal influences may pose a greater risk to a coach’s wellbeing due to the systemically entrenched nature of these factors. Bentzen and colleagues for instance, found that workload negatively predicted vitality and satisfaction with life in elite-level coaches [87]. Since excessive workloads have been identified as common stressors among elite-level coaches [8, 10, 100], organisations need to ensure that coaches are provided with manageable workloads to preserve their mental wellbeing (e.g. devolvement of responsibilities). As coaches are cognisant of the high demands that arise from the role, and usually acknowledge and accept the persistent job insecurity associated with the profession [88], organisations and federations must also establish ways to manage job insecurity and provide support to coaches who depart from their role or are relieved from their duties. This is critical as preliminary evidence indicates that job insecurity may negatively predict a coach’s hedonic and eudaimonic wellbeing [88], with job dismissals leading to emotional distress and negative affective states [24, 86]. Given these organisational factors tend to be structurally embedded [11], organisations and federations need to be mindful of the pressures they exert and the support they can provide in influencing an elite coach’s mental wellbeing. This support should also be extended to help address and manage broader cultural factors, as hypermasculine cultures for example, have been reported to impact the mental wellbeing of elite coaches, particularly among those who identify as women [85].

Understanding the wellbeing of elite-level coaches is important to both researchers and practitioners, as evidence suggests that low levels of wellbeing may increase the risk of experiencing mental ill-health over time [116–118]. There is some evidence to support this relationship in a coaching context, as Lee and Chelladurai found that positive affectivity negatively predicted emotional exhaustion, and negative affectivity positively predicted emotional exhaustion in NCAA Division I coaches [89]. Given the limited evidence base, future research would benefit from pursuing a broader and more rigorous evaluation of the relationships between wellbeing (e.g. hedonic and eudaimonic domains) and mental ill-health in an elite-level coaching context. For instance, researchers could replicate studies evaluating the mental health profiles of elite athletes, to examine whether coaches who are flourishing have greater protection against symptoms of mental ill-health than those  languishing [66, 119].

To date, an emphasis has been placed upon investigating burnout-related symptoms in elite-level coaches, highlighted by 50% of the included studies focusing on burnout. Symptoms were predominantly evaluated using the MBI, or components thereof (76%). It is recommended that future research should incorporate all dimensions of the MBI to accurately determine the severity and presentation of burnout dimensions in elite-level coaches [37].

Comparatively, 33% of the included studies investigated symptoms related to a variety of mental disorders (e.g. anxiety, depression). Given the paucity of symptom-level research, a greater body of evidence is needed to fully comprehend the extent to which elite-level coaches experience symptoms associated with mental disorders, and the array of factors that may influence the onset of a disorder. The prevalence of mental disorders among elite coaches is also poorly understood, as studies reporting prevalence rates are scarce, varied (e.g. rates of depressive symptoms range from 14 to 44%) and employ diverse methodologies (e.g. varying measures utilised and timing of assessments). In addition, all studies measuring the prevalence of mental ill-health registered quality appraisal scores of 60% or below. Common issues included low response rates (< 60%), low reliability (α < 0.70) and unvalidated measures [120]. In order to draw accurate and reliable comparisons across coaching samples and communities (e.g. elite athletes and the general population), researchers are encouraged to employ consistent and validated measures to foster methodological coherence (measures proposed by Gouttebarge et al. [121] may serve as useful starting point). It is also recommended that researchers should seek to evaluate the prevalence of mental ill-health using longitudinal study designs that incorporate larger sample sizes. The majority of prevalence studies were cross-sectional in nature (89%) and generally reported findings with less than 100 participants (67%), which risks reporting biases. In order to fully understand the extent to which elite-level coaches experience symptoms of mental ill-health, future research should seek to identify periods where coaches may experience heightened symptomatology, and also utilise more representative coaching samples (*n* > 100) [17].

Much like research examining wellbeing, numerous risk and protective factors operating at the individual, interpersonal, organisational and societal level were found to impact symptoms of mental ill-health in elite-level coaches. Commonly cited protective factors included effective psychological skills/emotional regulation, robust social supports and strong organisation or federation supports. Conversely, frequently referenced risk factors included excessive workloads and stress-related factors (e.g. proliferation of stressors). These findings however largely emerged from evidence examining burnout in elite-level coaches. Further research is subsequently needed to identify the prominent risk and protective factors that can make a coach susceptible or protected from the emergence of a mental disorder. Preliminary research for instance has found organisational stressors and daily life hassles positively predict symptoms of depression and anxiety in elite-level coaches [11, 111]. Although prospective research is needed to identify the range of factors that may influence coach mental health, future research should continue to adopt these quantitative approaches (e.g. regression models) to discern which factors significantly contribute to the presence or absence of a mental disorder. These findings may allow policymakers to generate supports and reduce threats that mitigate the onset of a mental disorder. Researchers are encouraged to employ a socioecological framework to examine risk and protective factors (see Olive et al. [39]), as this may help to establish interventions which adopt a systems level approach that specifically target the varying domains of influence.

Finally, when examining the relationship between mental health and coaching effectiveness, preliminary evidence indicates that an association may exist. Findings were operationalised through a coach’s own or athlete/team’s functioning, and largely implied that poor mental health contributed towards reduced coaching effectiveness. Since only 14% of the included studies explored themes associated with coaching effectiveness, research should seek to specifically examine this relationship in greater detail, due to the limited evidence base that predominantly sought to examine this relationship as a secondary line of inquiry or arose as an incidental outcome. Thelwell et al. for instance, investigated ways that stress experienced by elite-level coaches may impact the coach-athlete relationship (including coach effectiveness) [25]. Based on the findings, it was suggested that a coach's experience of stress could impact a coach’s psychological/emotional state (e.g. emotional regulation), standard of performance (e.g. organisational skills) and coaching style (e.g. changes in body language). In addition, stress was also found to shape an athlete’s psychological/emotional state (e.g. confidence), standard of performance (e.g. development) and behaviour (e.g. increased introversion). Since the coach-athlete relationship is argued to operate at the heart of coaching [122], further research is needed to explore how mental health outcomes could affect ways in which both coaches and athletes function. Given the multiple confounding variables that may influence a subjective construct like coaching effectiveness, it would also be valuable for future research to utilise validated measures to examine this relationship in further detail (e.g. the Coaching Behavior Scale for Sport; see Côté et al. [123] and Mallett and Côté [124]). The field would also benefit from exploratory qualitative research, to investigate the potential bi-directional relationship between a coach’s effectiveness and mental health.

Recognising the link between mental health and coaching effectiveness has important implications for the elite sports sector, as potential coach-related gains could be used to leverage broader interest and buy-in amongst elite-level coaches in consideration of their own mental health needs. If coaches become appreciative of the role mental health plays in their own or athlete/team’s functioning, coaches may seek to actively preserve and prioritise their psychological health. It is critical to ensure however, that individuals who are experiencing low wellbeing or mental ill-health are not discriminated against or ostracised from the elite sports environment. It is therefore important to be cognisant of the potential ramifications implied by this relationship, given that the desire to appear efficacious may reinforce stigma or reduce help-seeking in elite-level coaches.

### Limitations

There are limitations that may impact the utility of this review. Firstly, terms such as ‘coaching effectiveness’ and ‘elite’ are generally broad and ill-defined within the literature. This review’s inclusion criteria may have disregarded studies or excluded evidence that would have satisfied alternative definitions. Notwithstanding the challenges previously discussed, a unified set of definitions would generate a conceptual framework that promotes consistency and clarity throughout the field. Since the roles and responsibilities of athletes and coaches vary in elite-level environments, definitions that seek to address conceptualisations of ‘elite-level coaches’ should be cognisant of the differences between both populations. Moreover, despite efforts to group elite-level coaches together, it is important to recognize that an Olympic coach's experiences may vastly differ from an NCAA Division I coach for example. These role and pressure-related discrepancies should be considered when interpreting findings.

Other search-related limitations include the review’s language and time bias. Since the review sought to include studies that were published in English post-2000, the search strategy may have overlooked subsequent research outside of this criterion. Furthermore, given scoping reviews are considered to be descriptive and thematic in nature [49], it was beyond the scope of the review to perform a statistical synthesis. It is therefore suggested that the field would benefit from a future meta-analysis, that examined the prevalence of mental ill-health in elite athletes compared with the general population. Although technically a minimum of two studies are required to perform this analysis [125], given the small number of prevalence studies evaluating the proportion of elite-level coaches that experience specific outcomes associated with mental ill-health, it is suggested that the field would benefit from a future meta-analysis that is not limited to a small number of studies (< 5 studies) due to challenges associated with estimating the between-study variance [126]. Overall, further research should be undertaken to investigate levels of mental wellbeing and the proportion of elite-level coaches that experience mental ill-health at the population level.

Finally, due to the time sensitive nature of scoping reviews, only one reviewer was able to perform the quality appraisal. Consequently, given the MMAT’s broad criteria for assessment, it is suggested that a future systematic review should undertake a more rigorous quality appraisal to build upon the scoping review’s initial assessment. Although the MMAT helps to broadly assess the coherence and methodological robustness of the included studies [49], a more rigorous tool could build upon the review’s initial assessment and equip the field with a set of detailed and comprehensive insights (e.g. AMSTAR-2 or ROBIS) [127].

## Conclusion

Much like other populations in the elite sports environment, elite-level coaches are subject to varying experiences of wellbeing, and are susceptible to a number of symptoms associated with mental ill-health. Whilst research has predominantly examined themes related to burnout and wellbeing, less is known about the prevalence and manifestation of mental disorders in elite-level coaches. As a result, it is critical that researchers build upon the current clinical evidence base to fully comprehend the extent to which elite-level coaches experience mental disorders. To date, studies reporting the prevalence of mental ill-health in elite-level coaches are generally varied, inconsistent and often methodologically substandard. In order to address these issues, future research should ensure psychometric scales are reliable, validated and used consistently (e.g., all subscales). Such efforts will assist in accurate comparisons between populations and samples. Given 79% of studies registered quality appraisal scores of 60% or below, researchers should prioritise the development of high-quality methodologies that aim to enhance the strength of research within the field.

This review identified myriad risk and protective factors that operate at the individual, microsystem (interpersonal), exosystem (organisational) and macrosystem-level (societal). Since it was beyond the scope of the review to evaluate the impact of risk and protective factors, research is needed to determine which factors contribute most significantly towards mental health outcomes. Much like Longshore and Sachs [91] and Hägglund et al. [84], intervention studies should continue to explore the influence of protective factors such as psychological skills (e.g. resilience and self-reflection) and coping strategies (e.g. emotion-focused strategies), to protect and enhance the mental health of elite-level coaches due to the dynamism and malleability of these individual qualities. Despite the benefit of targeting interventions at the individual-level, from a sector perspective, organisations and federations should be cognisant of the role they play in preserving and influencing coach mental health. To reduce mental health pressures, organisational systems and structures must be prioritised to alleviate the burden placed upon elite-level coaches. For example, cultivating psychologically safe environments in elite sport may not only enhance the mental health of elite-level coaches [128], but extend to performance and organisational benefits (the latter including less staff turnover and associated financial costs). Organisations and governing bodies should also consider early intervention strategies (e.g. pathways to support) that aim to assist elite coaches who may be presenting symptoms prior to the emergence of a mental disorder, in order safeguard coaches from experiencing intensified symptoms that could lead to dismissal or resignation from the role [53]. Although current evidence indicates that multiple individual, interpersonal (e.g. athlete wellbeing) and organisational benefits emerge when coaches experience optimal mental health, ultimately, further high-quality research is required to better understand the mental health of elite-level coaches.

### Supplementary Information

**Additional file 1.** PRISMA-ScR Checklist.

**Additional file 2.** Example search strategy.

**Additional file 3.** Data extraction tool.

**Additional file 4.** MMAT results.

**Additional file 5.** List of references addressing the risk and protective factors that influence the mental health of elite-level coaches.

**Additional file 6.** List of references addressing the relationship between mental health and coaching effectiveness in elite-level coaches.

## Data Availability

All data and materials are freely available.

## Abbreviations

ASSQ: Athlete Sleep Screening Questionnaire
AUDIT-C: Alcohol Use Disorder Identification Tool-Concise
CBQ: Coach Burnout Questionnaire
CESD-R: Center for Epidemiologic Studies Depression Scale—Revised
DASS: Depression Anxiety Stress Scale
DSM-5: Diagnostic and Statistical Manual of Mental Disorders, 5th Edition
EAT-26: Eating Attitudes Test—26 items.
GHQ: General Health Questionnaire
IOC: International Olympic Committee
JBI: Joanna Briggs Institute
K-10: Kessler Psychological Distress Scale—10 items
MBI: Maslach Burnout Inventory
MBI-C: Maslach Burnout Inventory—Coach
MBI-ES: Maslach Burnout Inventory—Educators Survey
MBI-GS: Maslach Burnout Inventory—General Scale
MMAT: Mixed Methods Appraisal Tool
NCAA: National Collegiate Athletic Association Division I
PANAS: Positive and Negative Affect Schedule
PCC: Population-Concept-Context
PGSI: Problem Gambling Severity Index
PRISMA-ScR: Preferred Reporting Items for Systematic reviews and Meta-Analyses extension for Scoping Reviews
PROMS: Patient Reported Outcomes Measurement Systems
RESTQ: Recovery-Stress-Questionnaire
SEQ: Sport Emotion Questionnaire
STAI: State-Trait Anxiety Inventory
SVS: Subjective Vitality Scale
SWLS: Satisfaction with Life Scale
UWES: Utrecht Work Engagement Scale
WEMWBS: Warwick-Edinburgh Mental Well-being Scale

## Footnotes

1 Although sex and gender are considered as distinct constructs, to assist the interpretation and categorisation of findings sex and gender were merged to characterise the overall sample (e.g. male/men or female/women).
