# Supplementary material for: The Mental Health of Elite-Level Coaches: A Systematic Scoping Review
Source: Sports Med Open. 2024 Feb 11;10:16. doi: 10.1186/s40798-023-00655-8 (PMC10859359; doi:10.1186/s40798-023-00655-8)
Supplement: Supplementary file 2 — Additional file 2. Example search strategy. [file 40798_2023_655_MOESM2_ESM.docx]

Supplementary File 2: Example search strategy for PsycInfo.

| elite or high-performance or high performance or professional or olympic or paralympic or national or international or world-class or world class or college or collegiate or highly trained or competitive |
| --- |
| **AND** |
| mental disorders OR mental health OR stress* OR psychological distress OR suicide OR mental illness OR wellbeing OR well-being OR anxiet* OR anxious OR depress* OR phobi* OR burnout* OR burn-out* OR obsessive-compulsive OR panic OR eating disorder* OR anorexia OR anorexic OR bulimia OR bulimic OR binge eat* OR suicid* OR attention deficit hyperactivity disorder* OR attention deficit disorder with hyperactivity OR adhd OR bipolar OR psychotic* OR psychosis OR psychoses OR flourish* OR languish* OR thriving OR thrive OR alcohol* OR substance abuse OR substance dependence OR addiction OR sleep |
| **AND** |
| coach* or manager or director |
