# Supplementary material for: The Mental Health of Elite-Level Coaches: A Systematic Scoping Review
Source: Sports Med Open. 2024 Feb 11;10:16. doi: 10.1186/s40798-023-00655-8 (PMC10859359; doi:10.1186/s40798-023-00655-8)
Supplement: Supplementary file 3 — Additional file 3. Data extraction tool. [file 40798_2023_655_MOESM3_ESM.docx]

Supplementary File 3: Data extraction tool

| **Scoping Review Details** | | |
| --- | --- | --- |
| Scoping Review title: | The mental health of elite-level coaches: A systematic scoping review | |
| Review objective/s: | To identify what is known about the way in which high-performance sports coaches experience mental health. | |
| Review question/s: | 1. What are coaches’ experiences of wellbeing? 2. What is the nature and prevalence of mental ill-health in elite-level coaches? 3. What risk and protective factors influence coach mental health? 4. What are the various ways coaching effectiveness is conceptualised from a mental health perspective? 5. What is the relationship between mental health and coaching effectiveness? | |
| **Reasons for inclusion in scoping review** | | |
| Peer-reviewed journal | Yes/No | |
| After January 2000 | Yes/No | |
| Primary Research | Yes/No | |
| English language | Yes/No | |
| Full-text available | Yes/No | |
| Does the study collect data from elite or high-performance coaches who manage athletes at the Olympic, Paralympic, international, national, professional or NCAA Division I level? | Yes/No | |
| Does the study explore coaches who operate in positions of leadership or responsibility exclusively (e.g. head/assistant/senior coaches) or provide group findings separately where a heterogenous sample (e.g. strength & conditioning coaches) is utilised? | Yes/No | |
| Does the study explore coaches exclusively or provide group findings separately where a sample beyond coaches (e.g. athletes or support staff) is utilised? | Yes/No | |
| Does the study explore elite coaches exclusively or provide group findings separately where a heterogenous sample (e.g. elite and non-elite) is utilised? | Yes/No | |
| Does the study report on the mental health (mental wellbeing or mental ill-health) of elite coaches? | Yes/No | |
| **Evidence source Details and Characteristics** | | |
| Author |  | |
| Date article sourced |  | |
| Title |  | |
| Year of publication |  | |
| Journal |  | |
| Type of Study (e.g. qual, quant, cross-sectional) |  | |
| Study aims/objective |  | |
| Sample size | n = | |
| Response rate |  | |
| Location (of population) |  | |
| Participant details | Age |  |
|  | Sex | Male = (), Female = () |
|  | Sport |  |
|  | Individual/Team sport | Individual = (), Team = () |
|  | How is elite-level coaching defined? |  |
|  | Level of Coaching (e.g. Olympic, professional, national) |  |
|  | Type of Coach (eg. Head or Assistant) |  |
|  | Level of Engagement with Profession (e.g. Full-time/Part-Time/Voluntary) |  |
|  | Amount of Coaching Experience |  |
|  | Other |  |
| Outcome Measures | Wellbeing Measures (e.g. emotional, social, psychological) |  |
|  | Mental Ill-Health  Measures (e.g. anxiety, burnout, depression) |  |
| Conceptual Framework(s) (e.g. SDT, CMRT) |  | |
| Measures (e.g. interviews, wellbeing scales) |  | |
| Data Analysis (e.g. t-tests, thematic analysis) |  | |
| Context (e.g. preseason, post-season) |  | |
| **Details/Results extracted from source of evidence** | | |
| Results Summary | General results |  |
|  | Specific to wellbeing |  |
|  | Specific to mental ill-health |  |
|  | Specific to risk and protective factors |  |
|  | Specific to coach effectiveness |  |
|  | Specific to the conceptualisation of coach effectiveness |  |
| Key conclusions by authors |  | |
| Future research direction |  | |
| Funding body/sponsor |  | |
| Author identified limitations |  | |
| Additional limitations |  | |
| **Comments** | | |
|  | | |
