# Supplementary material for: The Mental Health of Elite-Level Coaches: A Systematic Scoping Review
Source: Sports Med Open. 2024 Feb 11;10:16. doi: 10.1186/s40798-023-00655-8 (PMC10859359; doi:10.1186/s40798-023-00655-8)
Supplement: Supplementary file 4 — Additional file 4. MMAT results. [file 40798_2023_655_MOESM4_ESM.docx]

Supplementary File 4: Mixed Methods Appraisal Tool (Hong et al., 2018)

| **Qualitative** | **1. Is the qualitative approach appropriate to answer the research question?** | **2.** **Are the qualitative data collection methods adequate to address the research question?** | **3. Are the findings adequately derived from the data?** | **4. Is the interpretation of results sufficiently substantiated by data?** | **5. Is there coherence between qualitative data sources, collection, analysis and interpretation?** |
| --- | --- | --- | --- | --- | --- |
| Baldock et al. [10] | Yes | Yes | Yes | Yes | Yes |
| Bentzen et al. [97] | No | Yes | Yes | No | No |
| Bentzen et al. [24] | Yes | Yes | Yes | Yes | Yes |
| Hägglund et al. [84] | No | No | Yes | Yes | Yes |
| Kenttä et al. [86] | Yes | Yes | Yes | Yes | Yes |
| Kenttä et al. [85] | No | No | Yes | Yes | Yes |
| Lundkvist et al. [100] | Yes | Yes | Yes | Yes | Yes |
| Olusoga et al. [13] | Yes | Yes | Yes | Yes | Yes |
| Olusoga & Kenttä [45] | No | Yes | Yes | Yes | Yes |
| Roberts et al. [110] | Yes | Yes | Yes | Yes | Yes |
| **Quantitative non-randomised** | **1. Are the participants representative of the target population?** | **2. Are measurements appropriate regarding both the outcome and intervention (or exposure)?** | **3. Are there complete outcome data?** | **4.** **Are the confounders accounted for in the design and analysis?** | **5.** **During the study period, is the intervention administered (or exposure occurred) as intended?** |
| Balk et al. [90] | No | No | Yes | Yes | Yes |
| Bentzen et al. [96] | Yes | No | No | Yes | No |
| Bentzen et al. [87] | Yes | No | No | Yes | No |
| Bentzen et al. [88] | Yes | No | No | Yes | No |
| Carling et al. [82] | No | No | No | No | Yes |
| de Sousa Pinheiro et al. [94] | No | Yes | Yes | No | Can’t Tell |
| Kellmann et al. [95] | Yes | Yes | Yes | No | No |
| Ruddock et al. [79] | Yes | Can’t Tell | Can’t Tell | Can’t Tell | Can’t Tell |
| Ruddock et al. [80] | Yes | Can’t Tell | Can’t Tell | Can’t Tell | Can’t Tell |
| **Quantitative Descriptive** | **1.** **Is the sampling strategy relevant to address the research question?** | **2.** **Is the sample representative of the target population?** | **3.** **Are the measurements appropriate?** | **4.** **Is the risk of nonresponse bias low?** | **5.** **Is the statistical analysis appropriate to answer the research question?** |
| Åkesdotter et al. [108] | Yes | No | No | Can’t Tell | Yes |
| Carson et al. [92] | Yes | No | Yes | No | Can’t Tell |
| Foretić et al. [109] | Can’t Tell | No | No | Yes | Yes |
| Gencay & Gencay [101] | Yes | No | No | Can’t Tell | Yes |
| Georgios & Nikolaos [102] | Can’t Tell | No | No | Can’t Tell | Yes |
| Hjälm et al. [99] | Yes | Yes | Can’t Tell | Yes | Yes |
| Kaski & Kinnunen [93] | Yes | No | No | No | Yes |
| Kegelaers et al. [11] | Can’t Tell | Yes | No | No | Yes |
| Kim et al. [111] | Yes | No | Can’t Tell | Yes | Yes |
| Lee & Chelladurai [89] | Yes | No | No | No | Yes |
| Lee [103] | Yes | No | Yes | No | Yes |
| Lundkvist et al. [104] | Yes | No | Yes | No | Yes |
| Nikolaos [105] | Can’t Tell | No | No | Can’t Tell | Yes |
| Pilkington et al. [12] | Yes | No | Can’t Tell | No | Yes |
| Ruddock et al. [78] | Can’t Tell | Can’t Tell | Can’t Tell | Can’t Tell | Can’t Tell |
| Ryska et al. [106] | Yes | No | No | No | Yes |
| Seo et al. [107] | Yes | No | No | Yes | Yes |
| Smith et al. [81] | Can’t Tell | No | Can’t Tell | Can’t Tell | Can’t Tell |
| Smith et al. [113] | Yes | No | Can’t Tell | No | Yes |
| Vinberg et al. [112] | Yes | No | No | No | Yes |
| **Mixed Methods** | **1.** **Is there an adequate rationale for using a mixed methods design to address the research question?** | **2.** **Are the different components of the study effectively integrated to answer the research question?** | **3.** **Are the outputs of the integration of qualitative and quantitative components adequately interpreted?** | **4.** **Are divergences and inconsistencies between quantitative and qualitative results adequately addressed?** | **5.** **Do the different components of the study adhere to the quality criteria of each tradition of the methods involved?** |
| Baldock et al. [83] | Yes | Yes | Yes | Yes | No |
| Hassmén et al. [98] | Yes | No | No | Can’t Tell | No |
| Longshore & Sachs [91] | No | No | Yes | No | No |
