# Supplementary material for: The Mental Health of Elite-Level Coaches: A Systematic Scoping Review
Source: Sports Med Open. 2024 Feb 11;10:16. doi: 10.1186/s40798-023-00655-8 (PMC10859359; doi:10.1186/s40798-023-00655-8)
Supplement: Supplementary file 5 — Additional file 5. List of references addressing the risk and protective factors that influence the mental health of elite-level coaches. [file 40798_2023_655_MOESM5_ESM.docx]

Supplementary File 5: List of references addressing the risk and protective factors that influence mental health in elite-level coaches.

| **Protective Factors** | | | **Wellbeing** | **Mental Ill-Health** |
| --- | --- | --- | --- | --- |
| Individual | Effective psychological skills or emotional regulation | 6 | - Hägglund et al. [84] - Kenttä et al. [86] - Longshore & Sachs [91] | - Georgios & Nikolaos [102] (Burnout) - Kegelaers et al. [11] (Anxiety/Depression) - Lee & Chelladurai [89] (Burnout – Emotional Exhaustion) - Longshore & Sachs [91] (Anxiety) |
|  | Effective coping strategies | 5 | - Baldock et al. [10] - Baldock et al. [83] - Bentzen et al. [88] | - Hjälm et al. [99] (Burnout) - Lee [103] (Anxiety) |
|  | Exercise | 2 | - Kenttä et al. [85] | - Hassmén et al. [98] (Burnout) |
|  | Coaching experience/age | 2 | - Nikolaos [105] (More experience was protective) | - Pilkington et al. [12] (Older age was protective) (Psychological Distress) |
|  | High levels of mental wellbeing | 2 |  | - Lee & Chelladurai [89] (Positive affect was negatively associated with burnout - emotional exhaustion) - Pilkington et al. [12] (Satisfaction with life balance was negatively associated with anxiety, depression, psychological distress and risky alcohol consumption) |
|  | Working full-time or part-time | 2 |  | - Hassmén et al. [98] (Working part-time was protective) (Burnout) - Hjälm et al. [99] (Working full-time was protective) (Burnout) |
|  | External non-coaching identities | 2 |  | - Hassmén et al. [98] (Burnout) - Lundkvist et al. [100] (Burnout) |
|  | High intrinsic and identified motivations | 1 |  | - Bentzen et al. [96] (Burnout – Emotional Exhaustion) |
|  | Collaborative Leadership style | 1 |  | - Ryska [106] (Burnout) |
|  | | | | |
| Microsystem | Strong social support | 8 | - Bentzen, et al. [88] - Kenttä et al. [85] - Kenttä et al. [86] | - Georgios & Nikolaos [102] (Burnout) - Lee [103] (Anxiety) - Nikolaos [105] (Burnout) - Olusoga & Kenttä [45] (Burnout) - Pilkington et al. [12] (Anxiety, Depression & Psychological Distress) |
|  | Engaging with a psychologist | 2 |  | - Hassmén et al. [98] (Burnout) - Olusoga & Kenttä [45] (Burnout) |
|  | | | | |
| Exosystem | Organisation and federation support | 6 | - Bentzen et al. [87] - Bentzen et al. [88] - Kaski & Kinnunen [93] | - Bentzen et al. [87] (Burnout) - Gencay & Gencay [101] (Burnout) - Hjälm et al. [99] (Burnout) - Roberts et al. [110] (Depression) |
|  | Sufficient recovery | 2 | - Kenttä et al. [85] | - Bentzen et al. [96] (Burnout – Emotional Exhaustion) |
|  | Reduced workload | 1 | - Kaski & Kinnunen [93] |  |
|  | | | | |
| Macrosystem | N/A | 0 |  |  |

| **Risk Factors** | | | **Wellbeing** | **Mental Ill-Health** |
| --- | --- | --- | --- | --- |
| Individual | Stress-related factors | 7 | - Baldock et al. [83] (Severity of stressors) - de Sousa Pinheiro et al. [94] (Perceptions of stress) - Olusoga et al. [13] (Perceptions of stress) | - Georgios and Nikolaos [102] (Perceptions of stress) (Burnout) - Kegelaers et al. [11] (Organisational stressors were positively associated with symptoms of anxiety and depression) - Kim et al. [111] (Quantity of stressors) (Depression) - Nikolaos [105] (Perceptions of stress) (Burnout) |
|  | Ineffective coping strategies | 3 | - Baldock et al. [10] - Baldock et al. [83] | - Baldock et al. [83] (Burnout) - Lee [103] (Anxiety) |
|  | Mental ill-health comorbidity (symptoms) | 3 |  | - Lee [103] (Subjective anxiety was positively associated with emotional exhaustion) - Ruddock et al. [78] (Emotional exhaustion was associated with depression, anxiety and psychological distress) - Ruddock et al. [79] (Emotional exhaustion was associated with depression, anxiety and psychological distress. Depersonalisation was associated with anxiety and psychological distress. Reduced personal accomplishment was associated with depression and psychological distress) |
|  | Coaching experience/age | 3 |  | - Bentzen et al. [97] (Lack of experience was a risk factor) (Burnout) - Gencay & Gencay [101] (More experience was a risk factor) (Burnout) - Pilkington et al. [12] (Younger age was a risk factor) (Psychological Distress) |
|  | Low autonomous motivation | 2 | - Bentzen et al. [87] | - Bentzen et al. [97] (Burnout) - Bentzen et al. [87] (Burnout – Cynicism & Reduced Personal Accomplishment) |
|  | Maladaptive Perfectionism | 2 |  | - Lundkvist et al. [100] (Burnout) - Olusoga & Kenttä [45] (Burnout) |
|  | Gender (women) | 2 | - Kenttä et al. [85] | - Kaski & Kinnunen [93] (Burnout) |
|  | Transitional phases | 2 | - Kenttä et al. [86] (Job termination) | - Kim et al. [111] (Retirement) (Depression) |
|  | Low levels of wellbeing | 1 |  | - Lee & Chelladurai [89] (Negative affect was positively associated with burnout - emotional exhaustion) |
|  | Low Emotional Intelligence | 1 |  | - Lee & Chelladurai [89] (Burnout – Emotional Exhaustion) |
|  | Controlling leadership style | 1 |  | - Ryska [106] (Burnout) |
|  | Dominant coaching identity | 1 |  | - Lundkvist et al. [100] (Burnout) |
|  | Family history of a mood disorder | 1 |  | - Kim et al. [111] (Depression) |
|  | Working full-time | 1 |  | - Kaski & Kinnunen [93] (Working full-time was a risk factor) (Burnout) |
|  | | | | |
| Microsystem | Lack of social support | 3 |  | - Georgios & Nikolaos [102] (Burnout) - Olusoga & Kenttä [45] (Burnout) - Nikolaos [105] (Burnout) |
|  | | | | |
| Exosystem | Excessive workload | 6 | - Bentzen et al. [87] | - Bentzen et al. [97] (Burnout) - Bentzen et al. [96] (Burnout – Emotional Exhaustion) - Bentzen et al. [87] (Burnout) - Hassmén et al. [98] (Burnout) - Lundkvist et al. [100] (Burnout) - Olusoga & Kenttä [45] (Burnout) |
|  | Lack of organisation or federation support | 4 | - Kenttä et al. [86] | - Bentzen et al. [97] (Burnout) - Gencay & Gencay [101] (Burnout) - Olusoga & Kenttä [45] (Burnout) |
|  | Lack of recovery | 4 | - de Sousa Pinheiro et al. [94] - Kellmann et al. [95] | - Bentzen et al. [96] (Psychological detachment and relaxation) (Burnout – Emotional Exhaustion) - Lundkvist et al. [100] (Burnout) |
|  | Poor work-life balance | 3 | - Kenttä et al. [85] | - Bentzen et al. [96] (Burnout – Emotional Exhaustion) - Olusoga & Kenttä [45] (Burnout) |
|  | Job insecurity | 2 | - Bentzen et al. [88] - Bentzen et al. [24] |  |
|  | Excessive organisational influence | 1 |  | - Bentzen et al. [97] (Burnout) |
|  | Sport type | 1 |  | - Kegelaers et al. [11] (Team sport was risk factor) (Risky Alcohol Consumption) |
|  | | | | |
| Macrosystem | Stigma towards help-seeking | 2 |  | - Olusoga & Kenttä [45] (Burnout) - Roberts et al. [110] (Depression) |
|  | Sporting culture | 2 | - Kenttä et al. [85] (Hypermasculine culture) | - Hassmén et al. [98] (Constant pressure to perform) (Burnout) |
|  | Media Scrutiny | 1 |  | - Olusoga & Kenttä [45] (Burnout) |
