# Supplementary material for: The Mental Health of Elite-Level Coaches: A Systematic Scoping Review
Source: Sports Med Open. 2024 Feb 11;10:16. doi: 10.1186/s40798-023-00655-8 (PMC10859359; doi:10.1186/s40798-023-00655-8)
Supplement: Supplementary file 6 — Additional file 6. List of references addressing the relationship between mental health and coaching effectiveness in elite-level coaches. [file 40798_2023_655_MOESM6_ESM.docx]

Supplementary File 6: List of references addressing the relationship between mental health and coaching effectiveness in elite-level coaches.

| **Coach Performance** | | | |
| --- | --- | --- | --- |
| Psychological/Emotional State | Focus | 3 | Lee [103]  Longshore & Sachs [91]  Lundkvist et al. [100] |
|  | Decision making | 1 | Olusoga & Kenttä [45] |
|  | Emotional regulation | 1 | Longshore & Sachs [91] |
|  | Confidence | 1 | Lundkvist et al. [100] |
|  | | | |
| Standard of Performance | Motivation | 1 | Olusoga & Kenttä [45] |
|  | Presenteeism | 1 | Seo at al. [107] |
|  | Work engagement | 1 | Balk et al. [90] |
|  | | | |
| Coaching Style | Verbal communication | 1 | Lundkvist et al. [100] |
|  | Leadership style | 1 | Lundkvist et al. [100] |

| **Athlete/Team Performance** | | | |
| --- | --- | --- | --- |
| Standard of Performance | Performance levels | 1 | Olusoga & Kenttä [45] |
